# End-of-Life Decision-Making Capacity in Older People With Serious Mental Illness

**DOI:** 10.3389/fpsyt.2021.752897

**Published:** 2021-09-22

**Authors:** Carla Kotzé, Johannes Lodewikus Roos, René Ehlers

**Affiliations:** ^1^Department of Psychiatry, Faculty of Health Sciences, School of Medicine, Weskoppies Psychiatric Hospital, University of Pretoria, Pretoria, South Africa; ^2^Department of Statistics, Faculty of Natural and Agricultural Sciences, University of Pretoria, Pretoria, South Africa

**Keywords:** end-of-life, decision-making capacity, values, elderly, serious mental illness

## Abstract

**Background:** The study's main aim was to assess the end-of-life decision-making capacity and health-related values of older people with serious mental illness.

**Methods:** A cross-sectional, observational study, was done at Weskoppies Psychiatric Hospital, Gauteng Province, South Africa that included 100 adults older than 60 years of age and diagnosed with serious mental illness. The Mini-Cog and a semi-structured clinical assessment of end-of-life decision-making capacity was done before a standardized interview, Assessment of Capacity to Consent to Treatment, was administered. This standardized instrument uses a hypothetical vignette to assess decision-making capacity and explores healthcare-related values.

**Results:** The Assessment of Capacity to Consent to Treatment scores correlated (*p* < 0.001) with the outcomes of the semi-structured decision-making capacity evaluation. Significant correlations with impaired decision-making capacity included: lower scores on the Mini-Cog (*p* < 0.001); a duration of serious mental illness of 30–39 years (*p* = 0025); having a diagnosis of schizophrenia spectrum disorders (*p* = 0.0007); and being admitted involuntarily (*p* < 0.0001). A main finding was that 65% of participants had decision-making capacity for end-of-life decisions, were able to express their values and engage in advance care discussions.

**Discussion and Conclusion:** Healthcare providers have a duty to initiate advance care discussions, optimize decision-making capacity, and protect autonomous decision-making. Many older patients with serious mental illness can engage in end-of-life discussions and can make autonomous decisions about preferred end-of-life care. Chronological age or diagnostic categories should never be used as reasons for discrimination, and older people with serious mental illness should receive end-of-life care in keeping with their preferences and values.

## Introduction

End-of-life care is associated with important decisions that can give rise to many ethical dilemmas and discussions (1). People with serious mental illness (SMI) have reduced life expectancies and higher rates of physical illness than the general population. Disparities between the health and health care for patients with schizophrenia and those without a diagnosis of a mental illness have been reported in the literature (2). This raises questions about the end-of-life care needs of this vulnerable population when they develop progressive disease with an expected survival of months or less (3, 4). Healthcare practitioners may neglect to discuss end-of-life care with patients out of a fear to provoke negative reactions or erroneous assumptions about how mental illness impairs healthcare decision making (5).

No specific psychiatric diagnosis is invariably associated with decisional incapacity, but some factors associated with decisional incapacity are unemployment, being diagnosed with schizophrenia or other psychotic disorders, poor insight, involuntary admission, lower cognitive scores, and older age (6). The goal of determining decisional capacity is to maintain a proper balance between respect for autonomy and protecting those who lack capacity from making harmful decisions. Informed consent can only be considered valid if a competent person is permitted to make a voluntary choice after disclosure of appropriate and sufficient information, in the absence of undue influence (7, 8).

Despite the development of several instruments, expert opinion is still considered the ideal for decision-making capacity (DMC) evaluations. Training and the use of a systemic approach while having a level of skepticism when performing these evaluations are recommended (9–11). The widely accepted four key components of DMC evaluations include understanding, reasoning, appreciation, and communicating a choice (12). DMC evaluation is an integral part of every interaction between a healthcare practitioner and a patient. Clinical judgment about DMC should take into consideration the history, clinical assessment, formal capacity measures, cognitive tests, and the experience of the evaluator. This should be applied to a specific situation and weighed against the possible risks associated with a decision (7, 13).

Another important factor that should be taken into consideration during these evaluations is how the values of the evaluator influences the value judgment that is made. Healthcare providers should be aware of this and should never impose their views on the patient (14). It should always be kept in mind that it would be unrealistic to expect extreme precision from a standardized instrument when it is applied to making complex, value-laden assessments of DMC (15). Provision should be made for patients to receive care that aligns with their values, highlighting the need to discuss end-of-life care with patients and their families to ensure that patient preferences are incorporated into advance care planning (16). Human rights considerations should include presumptions of DMC, consideration of advance care planning, and patient values (17).

The stigma associated with SMI remains, and at the end of life this can contribute to limited access or poor quality of care. Collaborative care should be developed to ensure equal access to end-of-life care (18). It should never be assumed that patients with SMI cannot make autonomous healthcare decisions, and the consideration of values is crucial in the process of advance care planning (19, 20). As the older population grows worldwide, the need for relevant research in end-of-life care has never been greater. The end-of-life preferences of older people with SMI are a neglected research area, especially in developing countries. We owe it to these vulnerable patients to explore their values, needs, and preferences with compassion and depth (21).

## Methods

This was a descriptive, cross-sectional, observational study conducted at Weskoppies Psychiatric Hospital, Gauteng Province, South Africa. The study population included mental healthcare users older than 60 years, receiving care at this hospital for SMI. SMI was defined as the presence of a mental illness of a duration of more than 2 years combined with serious functional impairment and 100 participants were included in total. In- and outpatients of all admission statuses according to the Mental Health Care Act 17 of 2002 (voluntary, assisted, or involuntary) were included. Purposive, homogenous sampling was used by identifying individuals older than 60 years of age from the hospital's database. Clinical files were checked for inclusion criteria (>60 years old; diagnosed with SMI; able to give informed consent to participate in the research or assent to participation with a surrogate/proxy decision-maker providing informed consent; able to read and write; able to communicate in English or Afrikaans at a limited working proficiency level; clinically stable enough to participate in prolonged interview). Patients diagnosed with a major neurocognitive disorder or intellectual disability, overtly aggressive patients unable to partake meaningfully in the interview, and patients with a current substance use disorder were excluded.

### Study Procedures and Measures

Data collection was done sequentially. After informed consent was obtained, socio-demographic, diagnostic, and treatment data were collected from clinical files. The most recent documented diagnoses according to the DSM-5 were captured (22).

Thereafter, the researcher administered a cognitive screen, the Mini-Cog (23). This screen was only used to add additional information for the evaluations to follow and participants were not excluded based on this screen, as advised by the creator of the tests (24). The sensitivity of the Mini-Cog ranges from 76 to 99% with a specificity range of 89–93% with 95% confidence interval (23, 25, 26). After administration of this brief screen, the researcher did a semi-structured 4-component clinical evaluation of end-of-life DMC and captured it as a dichotomous outcome. Provision was made for uncertain cases and for capturing a reason for impaired DMC.

The final assessment was the standardized Assessment of Capacity to Consent to Treatment (ACCT). The ACCT interview was developed by Moye et al. [(27), p. 40] after the research team reviewed existing instruments with the view to address the limitations that they identified in the existing scales, which included: “(1) to minimize the reliance on memory in the assessment of understanding; (2) to use multiple approaches for the assessment of reasoning and appreciation; (3) to incorporate the assessment of health care values into the determination of capacity” (27). An important consideration in the choice of this instrument to be used in the current study is the focus on values and preferences relevant to healthcare decision-making, including three key value domains. Firstly, it assesses the impact of treatment choices on valued activities and relationships. Secondly, it considers the individual's preferred decision-making style (autonomous, shared, deferred), and lastly, the views on quality vs. length of life. It also includes the influence of religious beliefs on the views expressed by the patient (27). A hypothetical vignette that describes a person who had a stroke. In this context, a subsequent decision had to be made for or against resuscitation in the event of cardiac arrest.

Some of the innovations in the ACCT include the assessment of understanding with cues to minimize memory demands and the assessment of appreciation with distrust and foresight subscales and expressing a choice is seen as a threshold ability. During the initial assessment of the reliability and validity of the ACCT, it was found to be good, with Cronbach internal consistency reliability of α = 0.96 based on all 56 items in their sample. The internal consistency reliability was α = 0.88 for the 18 items used in vignette two, the vignette that was used in the current study. A summary of the vignette details was used and the ACCT was administered according to the instructions provided by the creators (27). All anonymized data were captured in an Excel spreadsheet and exported to SPSS and SAS Statistics software for further analysis.

### Statistical Analysis

Descriptive statistics with frequencies and, where applicable, means and standard deviations (SD) were used for the sociodemographic information, primary psychiatric diagnosis, treatment groups, duration of SMI, Mini-Cog scores, DMC, and the results of the ACCT interview. In some of the data such as the valued activities, the distribution had low frequencies and no further sensible analysis could be done. The ACCT does not have clinical cut-off scores and is used as a tool to support professional clinical judgements. To allow sensible comparison of the ACCT scores with DMC outcomes, a Bonferroni correction was done for seven simultaneous pairwise comparisons, with means, SDs, median, and *p*-values. Spearman's rank-order correlation is a non-parametric test that measures the strength and direction of an association between two ranked variables, and it was used to evaluate the strength of association between the ratings on the Mini-Cog and ACCT sub-scores. Chi-square comparisons were done with some of the data, including DMC and some healthcare-related values. Testing was done at the 0.05 level of significance, unless specified differently. Multinomial logistic regression or logit model is frequently used when the dependent variable is nominal with more than two levels, and was used to describe the data and explain the relationship between DMC and specific independent variables.

## Results

### Descriptive Statistics

The age of the 100 participants ranged from 60 to 84 years (mean = 66.08; SD = 5.35). Refer to Table 1 for a summary of the sociodemographic information. The majority (66%) were following up as outpatients. The biggest group was voluntary outpatients (58%), followed by involuntary inpatients (25%), assisted inpatients (9%), and involuntary outpatients (8%). The most frequent diagnosis was schizophrenia spectrum and other psychotic disorders (61%). The other primary psychiatric diagnostic groups were major depressive disorder (20%) and bipolar disorder (19%), of which 16% were bipolar I and 3% bipolar II.

**Table 1:** Sociodemographic information of participants.

**Sociodemographic information**	**Frequency or %**	**Sociodemographic information**	**Frequency or %**
**Marital status:**		**Number of children:**	
Never married	26	No children	28
Married	21	1 child	14
Divorced/separated	32	2 children	29
Widow(er)	21	3 or more children	29
**Level of education:**		**Employment status:**	
Primary level	5	Unemployed	70
Secondary level	57	Medically boarded	13
Tertiary level	38	Retired pensioners	12
		Currently employed	5
**Home language:**		**Religion:**	
Afrikaans	37	Christian	85
English	33	Secular	12
Other languages	30	Other	3

Comorbidity was noted as psychiatric conditions (20%) and another medical condition (67%). A total of 233 different psychiatric treatments were prescribed, with antipsychotics prescribed the most (91; 39%), followed by antidepressants (59; 25.3%), mood stabilizers (31; 13.3%), and benzodiazepines (26; 11.2%). Other treatments included anticholinergics, thiamine, beta-blockers, anti-androgens, and three cases where electroconvulsive therapy was used in the past.

With the semi-structured DMC evaluation, 65% were found to have end-of-life DMC, 31% not to have DMC, and 4% to be uncertain. For the 31 participants that were found not to have DMC, the most common reason noted was cognitive impairment in 16 cases, with three of these cases also having lack of insight. In the remaining 15 cases, thought process disturbances (7%), delusional thinking (6%), and lack of insight (2%) were the main reasons noted. The reasons that were captured for those with uncertain capacity were cognitive impairment (2%), thought process disturbances (1%), and religious preoccupation that bordered on delusional thinking (1%). For further analysis and comparisons, the four uncertain cases were grouped with the 31 cases without DMC (*n* = 35), as the risks in end-of-life decisions are considerable and it would be a more cautious approach to classify them as not having DMC in high-risk circumstances.

### Healthcare-Related Values

Each participant had to choose three valued activities from a list to enable assessment of values-based reasoning. Further analysis of valued activities was not possible because of the low frequency in each cell. The chosen valued activities are summarized in Table 2. Living situations included long-term hospitalization (25%), care facilities (31%), living on their own (6%), and living with family or friends (38%). Valued relationships were with children (40%), siblings (28%), spouses/life partners (17%), friends (4%), parents (2%), and none (8%). With the importance of religion in healthcare decision-making, one participant was undecided, and the rest were completely or mostly influenced by religion (36%), somewhat influenced by religion (28%), or religion had no or limited influence (35%). Participants preferred equal shared decision-making with the doctor (58%) and with the family (38%). The odds were 2.74 times higher for participants with a psychotic disorder not to want their family involved with healthcare decisions when compared to participants with BD. The odds were 4.48 times higher for participants with a psychotic disorder to answer that they would want everything done to prolong their life if they were very sick, when compared to participants with BD. The odds for a participant who wanted to be resuscitated to choose to have everything done to prolong their life were 8.74 higher than for those who preferred not to be resuscitated.

**Table 2:** Valued activities chosen by participants.

**Valued activities**	**Frequency**	**%**
To take care of myself (e.g., bathing, dressing); not have to depend on others for help with daily life	55	18.3
To walk or move around by myself	28	9.3
To live at home	22	7.3
To think clearly about things	26	8.7
To make my own life decisions (e.g., about health, finances, housing)	45	15.0
To have relationships with family and friends	48	16.0
To practice my religion or spiritual life (faith, prayer)	47	15.7
To live without significant pain or discomfort	17	5.7
To do specific activities or hobbies that I enjoy	12	4.0

### Inferential Statistics

For further comparisons, the sub-scores of the ACCT were used. These include U1: Understanding disorder; U2: Understanding treatment; A1: Appreciation—Distrust; A2: Appreciation—Foresight; R1: Reasoning rational; R2: Reasoning values; C1: Expressing a choice (a threshold ability). The ACCT sub-scores and total were compared to DMC. A Bonferroni correction where the *p*-value was compared to 0.05/7 = 0.00714 was done since there are 7 simultaneous pairwise comparisons, as reflected in Table 3. For the Mini-Cog, a score of <4 can be used for greater sensitivity; the grouping was ≤ 3 for cognitive impairment and >4 for a normal cognitive screen (23). In a chi-square comparison of the Mini-Cog with impaired DMC, the mean was 1.89, SD = 1.231, and with DMC the mean was 3.72, SD = 0.976 with a *p* < 0.001. Non-parametric Spearman's rho assesses monotonic relationships, and for Mini-Cog and ACCT scores, the correlation was significant at the 0.01 level (2-tailed), as reflected in Table 4. Chi square comparisons of DMC with some of the sociodemographic and clinical factors were done. The only significant relationships were between DMC and level of education (Fisher exact test *p* = 0.0091); diagnosis (Fisher exact test *p* = 0.0007); admission status (Fisher exact test *p* < 0.0001); and duration of illness (Fisher exact test *p* = 0.0025).

**Table 3:** Comparison of ACCT sub-scores and outcome of semi-structured DMC evaluation.

	**Median**		***p*-value**
	**DMC No (*n* = 35)**	**DMC Yes (*n* = 65)**	
U1: Understanding disorder	1	4	<0.001
U2: Understanding treatment	3	6	<0.001
A1: Appreciation—Distrust	3	4	<0.001
A2: Appreciation—Foresight	1	3	<0.001
R1: Reasoning—Rational	1	3	<0.001
R2: Reasoning—Values	0	3	<0.001
C1: Expressing a choice	1	2	<0.001
**Total**	10	24	<0.001

**Table 4:** Spearman's rank order correlation between ACCT and Mini-Cog (*N* = 100).

**ACCT**	**Correlation**	***p*-value**
U1: Understanding disorder	0.526	<0.001
U2: Understanding treatment	0.477	<0.001
A1: Appreciation—Distrust	0.304	<0.001
A2: Appreciation—Foresight	0.361	0.002
R1: Reasoning—Rational	0.464	<0.001
R2: Reasoning—Values	0.514	<0.001
C1: Expressing a choice	0.431	<0.001
ACCT Total	0.552	<0.001

### Logit Model With DMC as Dependent Variable

A logit model was fitted with DMC as the dependent variable and the independent variables age, primary diagnosis, duration of illness, religion, level of education, marital status, home language, and admission status. Diagnostic categories and admission status regroupings were done because of low frequencies in cells. Bipolar and depressive diagnosis were grouped together as well as assisted and voluntary admission. Some of the variables were found not to be significant at the α = 0.01 level and were taken out of the model in a backwards elimination procedure. These variables were religion, home language, duration of illness, and marital status. The following variables remained in the model, and only two diagnostic categories (bipolar disorder and major depressive disorder combined and psychotic disorders separate) and two admission status categories (voluntary and assisted combined and involuntary admission separate) were significant at the 5% level. Refer to Table 5 for these findings.

**Table 5:** Logit model with DMC as the dependent variable.

**Type 3 Analysis of Effects**			
**Effect**	**DF**	**Wald Chi-Square**	**Pr > Chi-Square**
Age	1	3.0953	0.0785
Diagnosis	1	4.3024	0.0381
Level of education	1	3.2028	0.0735
Admission status	1	12.7311	0.0004
**Odds Ratio Estimates**			
**Effect**	**Point Estimate**	**95% Wald Confidence Limits**	
Age	0.910	0.820	1.011
Diagnosis	4.161	1.082	16.004
Level of education	0.345	0.108	1.107
Admission status	0.133	0.044	0.402

Keeping age, level of education, and admission status constant, the odds of a patient diagnosed with bipolar disorder or major depressive disorder are 4.161 times higher to have DMC compared to patients diagnosed with psychotic disorders. The 95% confidence interval for the odds ratio is 1.082, 16.004. Keeping age, diagnosis, and level of education constant, the odds of a voluntary or assisted patient are 7.519 (that is, 1/0.133) times higher to have DMC compared to an involuntary patient. The 95% confidence interval for the odds ratio is 2.488, 22.727. Keeping age, diagnosis, and admission status constant, the odds of a participant with a tertiary-level education is 2.899 times higher to have DMC compared to patients with only a primary- or secondary-level education. The *p*-value of 0.0736 and 95% confidence interval of 0.903, 9.259 indicate that this odds ratio does not differ significantly from 1. There is a tendency that the odds of DMC being present to decrease as age increases when keeping diagnosis, level of education, and admission status constant. However, this is not significant at the 5% level (*p* = 0.0785).

## Discussion

This study was the first quantitative study to assess the end-of-life decision making capacity and related values of older patients with SMI in the South African population. One prominent finding was the high rate of medical co-morbidity, with 67% of the participants having one or more medical comorbid condition. This is in keeping with the high morbidity rates and elevated mortality in patients with SMI. The health risks in this population are seen as a cumulation of socioeconomic problems, lifestyle factors, such as smoking and physical inactivity, and limited or delayed access to healthcare (28). Added to this, there is the increased risk for patients with SMI during the COVID-19 pandemic (29–31). Having a diagnosis of a mental disorder increased the risk of COVID-19 severity and mortality, and, specifically, patients with schizophrenia spectrum disorders have a considerable risk for mortality related to the SARS-CoV-2 virus (32, 33). Individuals with schizophrenia experience healthcare disparities and are especially at risk of having unmet needs at the end of life, with many barriers to access palliative care (34). In the current study, 61% of participants were diagnosed with schizophrenia spectrum and other psychotic disorders, emphasizing the urgent need for integrated care for this population. This pandemic should be used as a motivation to take collective action to ensure equitable access to health care for older adults with SMI (35).

With the semi-structured DMC evaluation done by the researcher, 65% of participants were found to have DCM for end-of-life decisions. When the ACCT sub-scores were compared to the DMC categories, with a Bonferroni correction for the 7 simultaneous pairwise comparisons, the medians were found to be significantly different in all cases. This significant correlation between the outcome of the clinical evaluation of DMC and the ACCT scores can be preliminary evidence of the usefulness of the ACCT as a tool to support professional clinical DMC assessments in this population of older patients with SMI. The creators of the ACCT found that cognitive and psychiatric symptomatology may influence the DMC differently, with possible impairment by paranoid ideas of the ability to appreciate the potential benefits of treatments. Executive dysfunction impaired the rational reasoning to balance the risks and benefits and to understand the provided information. From the current study, the same conclusion can be drawn: the ACCT may be suitable for detecting deficits in DMC when combined with a thorough clinical evaluation, including a cognitive evaluation and assessment of non-cognitive aspects that are relevant to the specific decision. The ACCT is especially useful in the psychiatric population because of the incorporation of a distrust component under the appreciation assessment that aims to identify individuals with a lack of insight into the seriousness of a condition or the potential benefits of treatment, and it can also identify possible paranoid ideas or even delusions toward the healthcare provider. The ACCT also includes two components of reasoning, with rational reasoning assessing the ability to compare risks and benefits. This is included in many approaches, but the ACCT adds the second subscale of reasoning to assess whether the individual can justify their choices as consistent with their expressed values. This is considered to be an important aspect to assess in the psychiatric population where thought process or content problems might prevent rational or values-based reasoning (27).

When the outcome of the DMC evaluation was compared to the results of the Mini-Cog, the finding was statistically significant (*p* < 0.001) for those with DMC having higher scores on the Mini-Cog. Out of the 31 participants who were found not to have DMC, cognitive impairment was the most common reason noted in 16 cases. This is in keeping with what has been reported in the literature (that neurocognitive functioning is more closely correlated with DMC than positive or negative symptoms in patients with schizophrenia). It was recommended that information should be provided repeatedly and cognitive remediation should be used to strengthen DMC (36). When there is too much focus on the cognitive aspect of decision-making, the under-emphasized non-cognitive aspects may be neglected. It is essential to always consider the values and emotions of the patient as well during these evaluations and to ensure that mental illness is not impairing the patient's DMC, even when they do not have cognitive impairment (10, 37). This recommendation is supported by the findings of the current study where the incapacity was judged to be due to delusional thinking, thought process disturbances, and lack of insight in cases without prominent cognitive impairments. The Mini-Cog can be recommended as a brief screening instrument that can provide valuable information to DMC evaluations, especially when there are time constraints. More extensive cognitive assessments can be requested in cases where DMC is unclear or cognitive impairment is identified.

Older adults may be more influenced in their decisions by their past experiences, than focusing just on the relevant facts (38). Age-related changes in cognition primarily involves executive functioning, verbal fluency, attention span, working memory, visuo-spatial memory processes, planning skills, difficulties with inhibition and overall slowing of cognitive processes. The changes in executive functioning with aging can be primarily explained by the reduction of processing speed and is dependent on the ability to recruit prefrontal circuits to compensate for performance difficulties. A posterior-anterior shift has been found with functional magnetic resonance imaging studies that has been postulated to be a compensatory mechanism. It was postulated to be a compensatory mechanism, because this shift if more pronounced when a task is new, complex and requires inhibitory control. When the stimulation is no longer new, the posterior-anterior shift is reduced, and this could possibly indicate that learning and the lifetime experience in decision-making and problem solving can compensate for the age-related decrease in processing speed. This should be taken into consideration especially in scenarios where new information is being discussed, by repeating information, clarifying terminology and providing sufficient time for the patient to make an autonomous decision (39). Supported decision-making is an emerging paradigm in which people get help from others to understand and address choices they encounter in everyday life. The aim is to increase self-determination, to empower individuals to make their own decisions as far as possible and to prevent the need for substitute decision-making (40, 41).

Statistically significant (*p* = 0.0091) findings included an association between having DMC and having a tertiary level education and are in keeping with what has been reported before (42, 43). In terms of the duration of SMI, only the group of 31 participants with 30–39 years' duration showed a statistically significant correlation (*p* = 0.0025) with having impaired DMC. This seems to be a novel finding that warrants further investigation as no studies about the duration of SMI and its relationship to DMC could be found. Most of the literature focus on the duration of untreated illness and the effect on prognosis or other outcome measures. Patients with schizophrenia have been found to have an increased risk of a dementia diagnosis and it has been found that people with younger age of mental disorder onset and more persistence of mental disorders, has accelerated aging in midlife, including impairments in sensory, motor and cognitive functioning (44, 45). Other statistically significant correlations with having impaired DMC were having a diagnosis of schizophrenia spectrum and other psychotic disorders (*p* = 0.0007) and being admitted involuntarily (*p* < 0.0001). From the logit model patients with schizophrenia spectrum disorders and involuntary admission were at a significantly higher risk not to have DMC, and this is in keeping with what has previously been reported (6, 42, 46, 47). Decisional incapacity in patients with psychotic disorders is responsive to interventions, such as simplification of the information, encouragement, and shared decision-making (46).

When some of the healthcare values were compared to DMC, nothing was found to be at the level of significance, but when compared with the diagnostic categories, the odds were found to be 4.48 higher for participants with schizophrenia spectrum and other psychotic disorders to want to prolong their life at all costs if they were ill, and 2.75 times higher not to want their families involved with healthcare decision, than for patients with BD. It was reported by Karel et al. (48) that patients who value quality of life over length of life are more likely to refuse life-sustaining treatments. The odds for a participant to prefer to be resuscitated and answer true to the statement about wanting everything done to prolong their life were 8.74 times higher than for those who preferred not to be resuscitated. This highlights how personal values can potentially predict choices about potential life-saving interventions.

Caring for elderly patients with SMI can pose many challenges, and these can be exacerbated by a life-threatening medical condition. To be able to provide optimal care for all patients with SMI, the obligation to do these evaluations cannot be placed solely with trained psychiatrists. All healthcare providers should be trained in DMC evaluations. Evidence-based approaches to optimize DMC should also be incorporated into training with a focus on the individualization of these evaluations. It should be done in a quiet environment with limited distractions, at opportune times when the patient is comfortable and well-rested. It is important to simplify and repeat information according to the patients' needs and to allow enough opportunity for gradual learning. Patients with slow processing speeds should not be pressured into making decisions and they should be allowed enough time to come to a decision without undue influence and pressure from families or healthcare providers. Encouragement and shared decision making are also considered essential. Training in and implementation of these measures to enhance autonomous DMC can ultimately reduce the stigma associated with SMI in older populations (39, 46).

Advance care planning is a process that supports adults at any stage of health in understanding and sharing their personal values, life goals, and preferences regarding future medical care (49). Advance care planning is seen as a way to facilitate supported decision-making, because it is a form of support for future decision-making (41). Policies about the model of care of this very vulnerable group of patients should address issues around end-of-life care and should encourage discussions with the patient and their family to elicit patient preferences and values. Routine implementation of advance care planning before urgent care is required and recommended. Cooperation and valid informed consent are always the goal, but in cases where this is not possible, having documented evidence of the patient's preferences and values can greatly assist the treating team to provide care that is in keeping with the patient's values. Discussions about end-of-life treatment preferences should be as detailed as possible and should be documented, preferably on an electronic platform that can be accessed by different healthcare providers. If such a system is not available, it should be done in an easily understandable format that the patient can take with them if they consult different healthcare providers or that families can easily access should the patient become incapacitated (4, 50).

Regardless of the DMC of a patient, transition to palliative care should always be an early consideration. Palliative care is an approach to end-of-life care that has been advocated by the World Health Organization and considers dying as a normal process. At the core of this approach is the focus on quality of life (51). There is also growing advocacy for following a palliative approach in psychiatric care, especially for treatment-resistant disorders where many of the typical interventions may be of a palliative nature. Following a palliative approach for patients with SMI with a limited functional prognosis and lifespan can potentially prevent therapeutic neglect and/or overly aggressive care. It also has the potential to improve quality of care, person-centeredness, prognosis, and autonomy for patients with SMI (52, 53).

Important aspects of palliative care include the clear communication of a diagnosis and prognosis, optimal symptom management, and support for advance care planning and for caregivers. Integration of psychiatric care and palliative care should be a priority for all facilities where long-term care for those with SMI is provided, and is an urgent consideration during the COVID-19 pandemic while these older people with SMI are considered especially at risk (53–56).

### Limitations and Recommendations for Future Research

The limitation of the cross-sectional design is that it does not take into consideration changes over time, an important consideration for future research. This study was specifically interested in the frequently neglected population of older people with SMI, and for this reason, homogenous, purposive sampling was considered appropriate. To address potential bias, inpatients and outpatients as well as patients of all legal admission statuses (voluntary, assisted, or involuntary) were included, but a limitation is that this study was only done at a single hospital, which limits the generalizability of the results. An area where some bias could have been introduced is the use of only a single researcher to perform all the assessments, but interrater reliability was not the focus of this study. The researcher has extensive training and experience with capacity assessments. The reasons why an additional rater was not included is because of the low interrater reliability that has been found with previous studies, the aim of this study was not the validation of a new instrument, and there is a lack of a gold standard to which to compare these assessments and instruments (57, 58). Due to this problem, it is not possible to comment on the validity of the DMC evaluations, except that there was a significant correlation with the ACCT assessment and the Mini-Cog scores, which is in keeping with previous findings of DMC. There is no full standardization for healthcare DMC given the contextual nature of these evaluations. Factors such as anxiety or fatigue can influence these assessments, and this might be problematic if different evaluations are done (59). The reliability and validity of capacity determinations can be improved through training and the addition of a structured instrument to the clinical assessment, especially when there is a focus on the specific legal standards for DMC such as the four components of decision-making. This type of systematic approach that has been recommended in the literature was preferred in this study (7, 11).

## Conclusions

It should be ensured that chronological age alone is never a reason for discrimination and that older populations with SMI retain the right to participate in healthcare decisions. If they do not have capacity for a specific decision, everything possible should be done to enhance their capacity and to ensure that any care provided is in keeping with their values and preferences. This study adds to the literature about end-of-life care in the older population with SMI, with an emphasis on the individual and contextual nature of DMC and the importance of the healthcare-related values of older patients with SMI. It is important to note that two thirds of older participants with SMI had DMC and were able to engage in end-of-life care discussions.

Policies should address the importance of DMC assessment and advance care planning in this vulnerable and often neglected group. Initiating end-of-life discussions and enquiring about the healthcare values of our patients can improve therapeutic relationships and the care provided to our patients, especially in settings where these discussions are not routinely implemented. Healthcare providers should initiate these discussions but should also be aware of those who lack DMC to be able to protect their rights and offer optimal opportunities to enhance DMC. Each patient should be offered the opportunity to discuss their healthcare values and preferences, with detailed documentation of these discussions to inform optimal end-of-life care for each individual, even during times when health resources are overstretched.

## Data Availability Statement

The raw data supporting the conclusions of this article will be made available by the authors, without undue reservation.

## Ethics Statement

The studies involving human participants were reviewed and approved by University of Pretoria's Faculty of Health Sciences Research Ethics Committee. The patients/participants provided their written informed consent to participate in this study.

## Author Contributions

CK formulated the research question(s), designed the study, collected the data, and wrote the article. JR was involved in a supervisory role and assisted with all the mentioned processes in that capacity. RE was the statistician involved with the study design, analysis of data, and write up of the article. All authors contributed to the article and approved the submitted version.

## Conflict of Interest

The authors declare that the research was conducted in the absence of any commercial or financial relationships that could be construed as a potential conflict of interest.

## Publisher's Note

All claims expressed in this article are solely those of the authors and do not necessarily represent those of their affiliated organizations, or those of the publisher, the editors and the reviewers. Any product that may be evaluated in this article, or claim that may be made by its manufacturer, is not guaranteed or endorsed by the publisher.
